# Is Mesh Becoming More Popular? Dilemmas in Urogynecology: A National Survey

**DOI:** 10.1155/2012/672356

**Published:** 2011-11-15

**Authors:** Alexander Condrea, Itamar Netzer, Shimon Ginath, Joseph Eldor-Itskovitz, Abraham Golan, Lior Lowenstein

**Affiliations:** ^1^Edith Wolfson Medical Center, 62 HaLohamim Street, P.O. Box 5, Holon 58100, Israel; ^2^Sackler Faculty of Medicine, Tel Aviv University, Tel Aviv, Israel; ^3^Department of Obstetrics and Gynecology, Ruth and Bruce Rappaport Faculty of Medicine, Technion-Israel Institute of Technology, Rambam Health Care Campus, 6 Ha'Aliya Street, P.O. Box 9602, Israel

## Abstract

The use of vaginal mesh in pelvic organ prolapse (POP) repair surgery has become more common in recent years. The purpose of the current study was to evaluate the common practice of Israeli urogynecologists, and to determine whether surgical practice has changed over the last two years. *Methods*. In 2009 and again in 2011, a survey was mailed to all urogynecologists affiliated with an academic institute in Israel. The survey consisted of 7 Likert-scale items and 3 open questions; the latter inquired about preferred type of surgery in three clinical scenarios. *Results*. Of 22 practitioners, 15 responded to the survey. The number of urogynecologists who reported using vaginal mesh for the repair of primary POP increased from 47 to 67% from 2009 to 2011. The number who would not use vaginal mesh in POP repair of elderly patients dropped from 60 to 3%. Finally, for the treatment of a 35-year-old patient with stage III uterine prolapse who desired to preserve fertility, 13% recommended the used vaginal mesh in 2009 compared with 47% in 2011. *Conclusion*. A survey of practitioners shows that the use of vaginal mesh for the repair of primary and recurrent pelvic organ prolapse has become more common among Israeli urogynecologists.

## 1. Introduction

The use of vaginal mesh in pelvic organ prolapse repair surgery has recently become more common [1]. A number of prolapse repair mesh devices have been designed by different companies and marketed extensively as a minimally invasive approach to pelvic floor repair. A Cochrane Collaboration review entitled “*Surgical management of pelvic organ prolapse in women,*” and based on 3773 patients in 40 trials, was published in 2010 [2]. The authors concluded that abdominal sacral colpopexy is associated with a lower rate of recurrent vault prolapse (RR 0.23, 95% CI 0.07–0.77) and dyspareunia (RR 0.39, 95% CI 0.18–0.86) than vaginal sacrospinous colpopexy, though the latter was found to have a shorter operating time. The use of mesh or graft inlays at the time of anterior vaginal wall repair was found to reduce the risk of recurrent anterior wall prolapse. Standard anterior repair was associated with more anterior compartment failures on examination than was polypropylene mesh repair as an overlay (RR 2.14, 95% CI 1.23 to 3.74) or armed transobturator mesh (RR 3.55, 95% CI 2.29 to 5.51) [2]. However, due to the paucity of peer-reviewed manuscripts, the authors advised relating to this procedure with caution. Reliable long-term data on the effect of vaginal mesh in pelvic organ prolapse surgery is particularly lacking.

 Subsequent to a metaanalysis of the use of vaginal mesh in pelvic organ prolapse repair surgery, the Society of Gynecologic Surgeons Systematic Review Group (SGS-SRG) issued two publications, “*Clinical practice guidelines on vaginal graft use*” [3] and “*Graft use in transvaginal pelvic organ prolapse repair, a systematic review*” by Sung et al. [4]. The objective of the former was to establish guidelines regarding the employment of synthetic grafts or native tissue repair in POP repair [3]. Weak evidence was found for the superiority of native tissue repair in anterior vaginal wall repair, when compared with biologic graft (4 trials). Weak evidence was also found for the superiority of native tissue repair in anterior vaginal wall repair, when compared with absorbable synthetic graft (2 trials) [3]. Moreover, weak evidence was found for nonabsorbable synthetic mesh improving anatomic outcomes of anterior vaginal wall repair, albeit with significant tradeoffs in regard to the risk of adverse events (2 trials). Regarding the superiority of native tissue repair for posterior prolapse versus absorbable synthetic graft or biologic graft, evidence was also weak (3 trials). Finally, no comparative studies were found that addressed the use of biologic grafts in multiple compartment repair compared with native tissue repair; the use of absorbable synthetic graft in multiple compartment vaginal wall repair compared with native tissue repair, or the use of nonabsorbable synthetic graft in multiple compartment repair compared with native tissue repair [3]. In conclusion, the authors noted that while the data supporting a lower rate of prolapse recurrence in graft use is limited, physicians should nevertheless consider and communicate to patients the seemingly improved durability of the procedure in the face of potential adverse events [4].

Due to the insufficient evidence, from a medical-legal point of view, the best course of action regarding the use of vaginal mesh in POP is a matter of debate. Some advocate that distinct informed consent be obtained for the use of vaginal mesh [5]. However, in a letter to the editor, Ann Weber states, “obtaining informed consent from patients for vaginal mesh placement during prolapse surgery cannot be achieved in light of the current dearth of data regarding risks and benefits [6],” and suggests that such procedures be regarded as “experimental.”

Despite the paucity of peer-reviewed studies on the use of vaginal mesh in pelvic floor prolapse repair, professional interest seems on the rise in recent years. A search of the PubMed database for the keywords “vaginal mesh” yields 118 articles published in 2009, 98 in 2010, and 53 as of July 2011, in all languages. These include randomized trials, basic science (e.g., ultrasound, histology, and animal models), case reports, retrospective series, guidelines, professional surveys, and reviews. 

The purpose of the current study was to evaluate the common practice of Israeli urogynecologists, and to evaluate trends in the practice of pelvic organ prolapse repair during the last two years.

## 2. Materials and Methods

An electronic survey was mailed to all fellowship-trained urogynecologists affiliated with an academic institute in Israel, in 2009 and again in 2011. The survey consisted of 7 Likert-scale score items and 3 open questions. Possible responses to the 7-point Likert-score questions ranged from “none of the time” to “all of the time.” Subjects included general mesh use; mesh use in light of comorbidity (diabetes, menopause, and stress urinary incontinence); and considerations of other factors (sexual activity, fertility). See The appendix for the full questionnaire. The open questions inquired about preferred type of surgery in three clinical scenarios. Participants were instructed not to consider financial factors in their decision making. Surveys were mailed back anonymously.

## 3. Statistical Analysis

SPSS for Windows version 18 (SPSS, Inc., Chicago, IL) was used for data management and statistical analysis. The chi-square test was used for comparison between dependent groups of categorical variables. All tests were considered significant at the  .05 level. All tests were 2 sided.

## 4. Results

The response rate of those who answered both in 2009 and 2011 was 68% (15/22). An increase in the number of urogynecologists who reported “frequently” or “almost always” using vaginal mesh for the repair of primary POP increased from 47 to 67%. Similarly, for recurrent POP, the number who would use vaginal mesh increased from 80 to 93%. For women older than 70 years, 60% of urogynecologists in 2009 compared with 33% in 2011 stated that they will rarely or never use meshes for POP repair. 

Regarding a case of a 55-year-old sexually active woman with uterine prolapse stage III, there was no change in the practice of the surveyed urogynecologists, with none of the participants choosing to perform abdominal or laparoscopic surgery, instead participants recommended vaginal hysterectomy and apical suspension with or without graft insertion. Regarding an 80-year-old healthy women with procidentia, only 13% chose to perform colpocleisis in 2009 compared with 47% in 2011 (*P* < 0.001). Finally, regarding the case of a 35-year-old patient with stage III uterine prolapse who desired to preserve fertility, 2 (13%) recommended Manchester surgery with the insertion of vaginal mesh in 2009 while 7 (47%) recommended the use of mesh in 2011, with only one of them recommending Manchester surgery (*P* < 0.001).

## 5. Discussion

This survey shows an increasing trend in the use of vaginal mesh for pelvic organ prolapse (POP) repair by Israeli pelvic floor surgeons over the last two years. This was apparent for primary and recurrent POP repair, as well as for POP repair in patients presenting with concomitant disease, menopause, or lifestyle considerations. Importantly, in women desiring preservation of fertility, there appears to be a marked increase in the use of mesh in hysteropexy, which may reflect on increase in documentation of favorable results pertaining to pregnancy [7, 8]. The reported increase in the use of colpocleisis may result from current training of physicians, and increased caution by urogynecologists regarding possible mesh complications in the elderly population.

The use of vaginal meshes in POP repair has increased in Israel despite the lack of randomized controlled trials supporting such use, and despite seemingly unresolved legal complications regarding the extent of patients' consent. The latter issue is not merely a technicality—long-term stability and risks of complications from these procedures are as yet unknown. Nevertheless, the reasons for the growing popularity of vaginal mesh are varied. First, Israeli surgeons practice medicine in an environment characterized by innovation and scientific progress, exemplified by Israel's reputation as a world leader of biotechnological research and development. Second, the boon of new mesh products, accompanied by powerful marketing efforts, has made a wide array of vaginal mesh products available to surgeons. Third, experience and mastery of the use of vaginal meshes may alleviate previous reservations in favor of the new technology. Fourth, and perhaps of prime importance, the ease of use of the new mesh products, along with physicians' own experience about better durability in POP repair with mesh compared with native tissue, is making them lucrative for most gynecologists. 

The use of a nonvalidated questionnaire is a limitation of the current study. Further, its anonymity precluded assessment of such characteristics of urogynecologists as number of years in practice and place of training. Most importantly, we have no data regarding the number of vaginal mesh procedures that were actually performed by each gynecologist, which may constitute a reporting bias on the part of the respondents.

## 6. Conclusion

The survey reported herein demonstrates a recent increase in the popularity of vaginal mesh use for the repair of pelvic organ prolapse among Israeli urogynecologists.

Randomized controlled trials of the use of vaginal mesh for POP repair are needed to determine optimal indications for their use. In the meantime, caution should be advised in the application of this yet unproven technology [4].

## Appendix

### Survey Questionnaire

Questions 1–3 pertain to women referred due to stage 3 prolapse of at least one compartment.

**Table:**

	Always					Never
	5	4	3	2	1	0

(1) Use of vaginal mesh in primary POP repair	5	4	3	2	1	0
(2) Use of vaginal mesh in recurrent POP repair	5	4	3	2	1	0
(3) Would you use a vaginal mesh in a patient who is disinterested in sexual intercourse?	5	4	3	2	1	0
(4) Would you use a vaginal mesh in women over 70 years of age?	5	4	3	2	1	0
(5) Do you consider insulin dependent diabetes mellitus a contraindication to vaginal mesh use?	5	4	3	2	1	0
(6) In a menopausal patient with a stage III prolapse of one vaginal wall and stage II uterine prolapse, would you prefer to *preserve* the uterus?	5	4	3	2	1	0
(7) In a menopausal patient with stage I uterine prolapse and stage III elongation of uterine cervix, would you prefer to preserve the uterus?	5	4	3	2	1	0
(8) A healthy, physically, and sexually active 55 year old. Diagnosis: stage III uterine prolapse, stage III cystocele, gaping introitus, stress urinary incontinence.						
Your choice of procedure: …………………………						
(9) A healthy 80-year-old patient who is not sexually active. Diagnosis: total prolapse.						
Your choice of procedure: …………………………						
(10) A 35-year-old woman desiring preservation of fertility. Diagnosis: stage III uterine prolapse, stage II cystocele, stage II rectocele (pessary treatment failed).						
Your choice of procedure: …………………………						

## References

[1] Feiner B, Jelovsek JE, Maher C (2009). Efficacy and safety of transvaginal mesh kits in the treatment of prolapse of the vaginal apex: a systematic review. *International Journal of Obstetrics and Gynaecology*.

[2] Maher C, Feiner B, Baessler K, Adams EJ, Hagen S, Glazener CM (2010). Surgical management of pelvic organ prolapse in women. *Cochrane Database of Systematic Reviews*.

[3] Murphy M (2008). Clinical practice guidelines on vaginal graft use from the society of gynecologic surgeons. *Obstetrics and Gynecology*.

[4] Sung VW, Rogers RG, Schaffer JI (2008). Graft use in transvaginal pelvic organ prolapse repair: a systematic review. *Obstetrics and Gynecology*.

[5] Mucowski SJ, Jurnalov C, Phelps JY (2010). Use of vaginal mesh in the face of recent FDA warnings and litigation. *American Journal of Obstetrics and Gynecology*.

[6] Weber AM (2010). Informed consent cannot be obtained for use of vaginal mesh. *American Journal of Obstetrics and Gynecology*.

[7] Hefni M, El-Toucky T (2010). Uterine prolapse in young women. *Bailliere’s Best Practice and Research*.

[8] Ridgeway B, Frick AC, Walter MD (2008). Hysteropexy: a review. *Minerva Ginecologica*.

